# Hands Up! Atypical Defensive Reactions in Heavy Players of Violent Video Games When Exposed to Gun-Attack Pictures

**DOI:** 10.3389/fpsyg.2019.00191

**Published:** 2019-02-05

**Authors:** Maria Fernanda Santos, Aline F. Bastos, Jose M. Oliveira, Ivan Figueira, Sonia Gleiser, Mirtes G. Pereira, Eliane Volchan, Fátima S. Erthal

**Affiliations:** ^1^Institute of Biophysics Carlos Chagas Filho, Universidade Federal do Rio de Janeiro, Rio de Janeiro, Brazil; ^2^Institute of Psychiatry, Universidade Federal do Rio de Janeiro, Rio de Janeiro, Brazil; ^3^Biomedical Institute, Universidade Federal Fluminense, Niterói, Brazil

**Keywords:** violent games, posturography, defensive reactions, PTSD, immobility, video games, gun, emotion

## Abstract

Threatening cues and surrounding contexts trigger specific defensive response patterns. Posturography, a technique for measuring postural strategies, has been used to evaluate motor defensive reactions in humans. When exposed to gun pointed pictures, humans were shown to exhibit an immobility reaction. Short and long-term exposure to violent video games was shown to be a causal risk factor for increased violent and aggressive behavior. Assaultive violence with a gun is a major trigger for motor defensive reactions, and post-traumatic stress disorder (PTSD) is the most characteristic psychiatric sequelae. Recent studies point to links between PTSD symptoms and emotional shortfalls in non-clinical trauma-exposed samples. The present study investigated defensive reactions to gun threat and PTSD symptoms in heavy players of violent video games compared to non-players. Male university students were screened according to use of violent video games and divided in three groups: non-players, moderate players, and heavy players. Stimuli were pictures depicting a man pointing a gun directed at the participant. In matched control pictures, non-lethal objects replaced the gun. Posturography was recorded and PTSD symptoms were assessed. When exposed to the threat pictures, non-players exhibited the expected reduction in amplitude of body sway (immobility), heavy players presented atypical augmented amplitude of body sway, and moderate players showed intermediate reactivity. Heavy players presented a significant distinct reaction compared to non-players. They also scored significantly higher in PTSD symptoms than non-players. Disadvantageous defensive reactions and higher vulnerability to PTSD symptoms, revealed in the present study, add to other shortcomings for heavy players.

## Introduction

“Violence is an extreme form of aggression that has the potential to produce severe physical harm, such as injury or death, to another” (Anderson et al., 2017, p. 143). Interpersonal violence involving guns has increased across the world (WHO, 2014; Grinshteyn and Hemenway, 2016), and populations living in large urban centers are specially affected (Ribeiro et al., 2013; Fowler et al., 2015). Assaultive violence with a gun is a major trigger for motor defensive reactions in humans, and posttraumatic stress disorder (PTSD) is the most characteristic psychiatric sequelae. Patients with PTSD were shown to be more susceptible to the hazardous effects of repeated activation of stress mediators on dysregulation of brain and body allostasis (McEwen and Gianaros, 2010). Recent studies point to links between PTSD symptoms and emotional shortfalls in non-clinical trauma-exposed samples (Miles et al., 2017).

The Global Status Report on Violence Prevention (WHO, 2014) proposed adopting a number of preventive strategies. Besides the reduction of access to guns and knives, changing of cultural and social norms that support violence was stressed. Guns figure prominently in the culture of violence, with a heavy contribution from the media (Smith et al., 2004). Indeed, “violence in screen entertainment media (i.e., television, film, video games, and the Internet), defined as depictions of characters (or players) trying to physically harm other characters (or players), is ubiquitous” (Anderson et al., 2017, p. 142).

Extensive research has shown that media violent content is a causal risk factor for increased violent and aggressive behavior (Bender et al., 2018). Playing video games, more than passively watching violent TV and films, has an even greater effect. Exposure to violent scenes in video games occurs in a more active context and the player is rewarded by acting violently (Anderson and Gentile, 2014).

Through posturography, a technique to study body sway, distinct defensive reactions were revealed (Volchan et al., 2017). Bastos et al. (2016) recorded defensive reactions to pictures depicting realistic gun attack and showed a reduction in amplitude of body sway indicating an immobility reaction to pictures of men pointing guns straight at the participant. The authors excluded heavy players of violent video games from the analyses, considering the literature on their proneness to aggression (Anderson et al., 2010; Greitemeyer, 2014).

Given the importance of studying the hazardous effects of exposure to violent video games, the present study investigated if those heavy players of violent video games present atypical defensive reactions to gun threat and whether they show differences in PTSD symptoms compared to non-players.

## Materials and Methods

### Participants

The database for the present study derived from Bastos et al. (2016). The sample consisted of 88 (48 men) undergraduate and graduate students who reported no neurological, psychiatric, or orthopedic disorders and were not under medications with nervous system action. The Ethics Institutional Review Board of the Federal University of Rio de Janeiro approved the study and participants provided written informed consent.

### Questionnaires

#### Frequency of Violent Video Game Play

Participants had to answer the following question “Do you play video games with violent contents (people using firearms)?”. The alternatives were “never”, “sometimes”, “often”, and “almost always”.

#### Posttraumatic Stress Symptoms

Participants completed the Trauma History Questionnaire (Green, 1996; Fiszman et al., 2005). This questionnaire lists life-threatening events. Participants indicated the event they considered the most intense and completed the PTSD Checklist for DSM-IV (PCL-C for DSM-IV) (Weathers et al., 1993; Berger et al., 2004) based on this event. The PCL-C for DSM-IV is a 17-item self-report measure of the severity of symptoms. Using a 5-point Likert scale (1 = *not at all*, 5 = *extremely*), participants rated the extent to which each symptom has disturbed them in the past month, providing a total symptom score and a score for each cluster (re-experiencing, avoidance and numbing, and hyperarousal). Participants scoring above the cutoff for clinical PTSD were excluded. Thus, in the sample of 88 participants, none reached the criterion for possible PTSD.

### Visual Stimuli

Sixteen pictures showed a man pointing a gun directed toward the participant (threat set). Sixteen other pictures (control set) showed, instead of a weapon, a man carrying a non-lethal object directed toward the participant. Within each set, individual pictures were presented for three seconds with no interval between them. Each set lasted for 48 s and the control set preceded the threat set. See Supplementary Material for more details.

### Data Collection

Details of posturographic recordings are described in Bastos et al. (2016). Briefly, amplitude of body sway was estimated by recording center of pressure displacement using a force platform. Participants stood motionless on the force platform looking at the presentation monitor while control and threat pictures were presented and posturographic signals were recorded. The posturographic parameter analyzed here was the standard deviation of center of pressure displacement (amplitude of body sway) in the anterior-posterior direction.

### Data Analysis

The difference in the anterior-posterior amplitude of sway during exposure to threat and control sets of pictures defined a reactivity index (Δ = *threat – control*). Total scores on the PCL-C for DSM-IV and scores in the three clusters (hyperarousal, reexperiencing, and numbing/avoidance) were computed.

Statistical analyses were performed with Kruskal-Wallis H and Mann-Whitney U tests. The threshold for significance was *p* < 0.05.

## Results

### Frequency of Violent Video Game Play

Participants were grouped according to the frequency of violent video game play. Participants reporting never playing violent video games were classified as “non-players” and the ones who reported playing “sometimes” were classified as “moderate players”. “Heavy players” were those that reported using violent video games “often” or “almost always”. The distribution of the three groups separated by gender is presented in Table 1.

**Table 1:** Distribution of the three groups separated by gender.

GROUP GENDER	Non-players	Moderate players	Heavy players
Male	15	21	12
Female	24	11	5


To avoid bias due to the very different proportion of men and women in each group and the low number of heavy players who were women, the analyses focused on men.

The final sample of 48 men had age (23.5 ± 4.11 y.o.) and weight (70.1 ± 11.17 kg) evenly distributed among the three groups (Age: *F*(2,45) = 0.90, *p* = 0.40; weight: *F*(2,45) = 0.36, *p* = 0.70).

### Posturography

**FIGURE 1:**
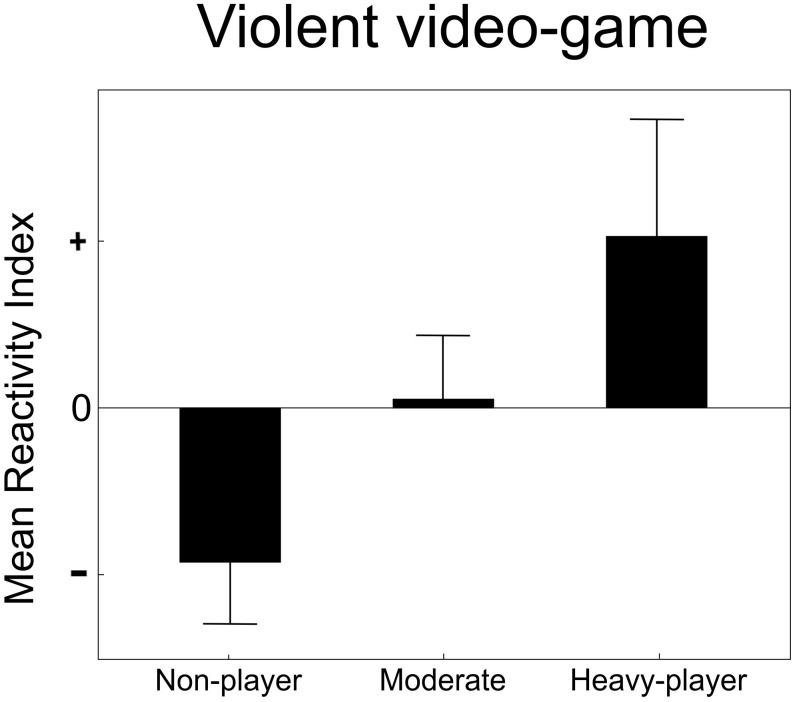
Exposure to violent video games and defensive reactions to pointing gun pictures. Anterior-posterior amplitude of body sway during presentation of threat (gun) minus control (non-lethal object) pictures, the reactivity index, is displayed according to participants’ frequency of violent video game play. Bars illustrate means and standard errors. Non-players exhibit reduced anterior-posterior amplitude of sway (immobility), heavy players exhibit a reverse reaction (augmented anterior-posterior amplitude of sway), and moderate players exhibit intermediate reactivity.

Figure 1 illustrates the reactivity index (*threat – control*) associated with the anterior-posterior amplitude of body sway for the three groups. It can be observed that non-players exhibited a reduction in amplitude of body sway (negative reactivity index), heavy players presented an augmented amplitude of body sway (positive reactivity index), and moderate players showed intermediate reactivity.

Analysis of the reactivity index revealed a significant main effect for group (*H*(2,48) = 7.39, *p* = 0.02). Follow-up *post hoc* analyses showed a significant difference between the reactivity of non-players and heavy players (Z = -2.56, *p* = 0.01). Heavy players presented a distinct reaction compared to non-players.

### PTSD Symptoms

Given the postural differences between heavy players and non-players, scores on the PTSD symptoms scale (PCL-C for DSM-IV) were compared between the two groups. Participants who anchored their PCL-C responses on a life-threatening trauma were included in the present analysis (14 non-players and 8 heavy players).

Total scores on the PCL-C were significantly higher for heavy players compared to non-players (Z = -2.06, *p* = 0.04). Scores on the PCL-C hyperarousal cluster were also significantly higher for heavy players (Z = -2.08, *p* = 0.04). Scores on the numbing/avoidance cluster, although higher for heavy players, did not reach statistical significance (Z = -1.77, *p* = 0.08). Scores on the reexperiencing cluster did not differ (Z = 0.78, *p* = 0.41).

The difference in PTSD symptoms between heavy players and non-players remained statistically significant (PCL-C total score (*Z* = 2.58, *p* = 0.01)) when participants who did not anchor their PCL-C responses on a life-threatening trauma were included in the analysis.

## Discussion

Previously, we studied defensive reactions in a sample that did not include heavy players (Bastos et al., 2016). That work showed a reduction in anterior-posterior amplitude of sway under exposure to threatening pictures depicting a man pointing a gun at the participant. Here, non-players presented this expected immobility reaction. Heavy players showed, as hypothesized, an atypical reaction (augmented anterior-posterior amplitude of sway). Furthermore, heavy players presented higher scores on the PTSD symptoms scale.

### Motor Defensive Response Patterns

Across species, threatening cues and surrounding contexts trigger specific defensive response patterns. Motor reactions, either overt actions or immobility, are the core of the observable defensive behaviors. Research on rodents (Blanchard and Blanchard, 1971, 1989; Blanchard et al., 1986) showed that, under attack by a predator or co-specific, flight is the dominant response when escape is available. When escape routes are blocked, immobility ensues. This action inhibition is considered an evolutionary adaptive strategy embedding preparatory states for action to be released only when appropriate context conditions trigger an action shift, like jump-attack seen in rodents (Blanchard et al., 1986).

In the present study, an attack-like condition was created by the presentation of pictures depicting a man pointing a gun at the participant. Non-players exhibited an immobility-like reaction. Heavy players behaved as if “jumping the gun”, increasing mobilization, instead of immobilizing and waiting for the best chance to get rid from danger. Indeed, “... the simple sight of a weapon increases the likelihood of aggression if the person has mentally paired a weapon such as a gun with killing or hurting people...” [Anderson and Warburton (2012), p. 72]. This is the case for heavy players of violent video games who are frequently exposed to weapon use for the purpose of killing and hurting others. The contrasting reaction of heavy *vs.* non-players to attack-like pictures add to converging evidence showing that exposure to violent video games is a causal risk factor for increased violent and aggressive behavior [for meta-analyses, see Anderson and Bushman (2001), Anderson et al. (2010), Bushman and Huesmann (2006), Greitemeyer and Mügge (2014)]. No previous study had employed posturography to investigate reactions of heavy-players to gun attack pictures.

### PTSD Symptoms

There are several potential drawbacks associated with a high frequency of violent video game playing. Heavy exposure to violent video games can lead people to see the world as more dangerous, to be more fearful, and initiate more self-protective behaviors such as carrying guns (Anderson and Gentile, 2014). Several studies have shown that civilian gun ownership, rather than protecting, increases the risk of both perpetrating and being a fatal victim of violence (Bangalore and Messerli, 2013; Branas et al., 2009; Cukier and Eagen, 2018; Monuteaux et al., 2015).

Heavy players scored higher than non-players on the PTSD symptoms scale, especially on the hyperarousal cluster, which includes feeling irritable or having angry outbursts, being “super-alert” or watchful or on guard, and feeling jumpy or easily startled. Symptoms such as angry outbursts may be related to the results of numerous studies that point to the effects of violent video games on increasing aggressiveness (Anderson et al., 2010; Anderson and Gentile, 2014; Greitemeyer and Mügge, 2014). Being on guard and feeling jumpy, along with angry outbursts, are consistent with heavy players adopting a precipitated (and possibly disadvantageous) response to an attack-like context.

## Limitations

This is a secondary analysis of previously collected data. The experiment was originally designed to investigate basic defensive reactions in humans. A convenience sample was recruited, ending up with a relatively small proportion of heavy players of violent video games. Characterization of heavy players was based on the high use of violent video games depicting guns, whereas other forms of violent content were not assessed. Furthermore, a standard validated questionnaire was not employed. The sample was not screened by trait questionnaires that assess aggressiveness. Finally, the present research is based on a cross-sectional sample and does not allow conclusions to be made regarding causality.

## Conclusion

Accumulating evidence led to a clear consensus that a high frequency of exposure to violent video games significantly alters important interpersonal behaviors in negative ways (Bender et al., 2018). Atypical disadvantageous defensive reactions and higher vulnerability to PTSD symptoms, revealed in the present study, add to other shortcomings for the heavy players themselves.

## Ethics Statement

This study was carried out in accordance with the recommendations of Declaration of Helsinki. The protocol was approved by the Ethics Institutional Review Board of the Federal University of Rio de Janeiro. All subjects gave written informed consent.

## Author Contributions

MS, AB, JO, SG, MP, EV, and FE designed the research. MS, AB, JO, IF, EV, and FE performed the research and analyzed data. AB, JO, SG, EV, and FE wrote the paper. All authors reviewed draft versions and approved the final version.

## Conflict of Interest Statement

The authors declare that the research was conducted in the absence of any commercial or financial relationships that could be construed as a potential conflict of interest.
