# Supplementary material for: Hands Up! Atypical Defensive Reactions in Heavy Players of Violent Video Games When Exposed to Gun-Attack Pictures
Source: Front Psychol. 2019 Feb 5;10:191. doi: 10.3389/fpsyg.2019.00191 (PMC6370668; doi:10.3389/fpsyg.2019.00191)
Supplement: Supplementary file 1 [file Data_Sheet_1.PDF]

Supplementary Material

Hands Up! Atypical Defensive Reactions in Heavy Players of Violent Video Games When Exposed to Gun-Attack Pictures

Maria Fernanda Santos, Aline F. Bastos, Jose M. Oliveira, Ivan Figueira, Sonia Gleiser, Mirtes G. Pereira, Eliane Volchan*, Fátima S. Erthal

*** Correspondence:** Eliane Volchan: elivolchan@gmail.com

1. Visual Stimuli

Sixteen pictures showed a man pointing a gun directed toward the participant (threat set). Sixteen other pictures (control set) showed, instead of a weapon, a man carrying a non-lethal object directed toward the participant. All pictures were acquired from web (www.gettyimages.com), taken by a professional photographer in a studio and from the International Affective Pictures System (Lang et al., 2005). Threat and control sets were matched in brightness, contrast and spatial frequency. The stimuli were presented on a 37in LCD monitor.

2. Posturography

Amplitude of body sway was estimated by recording center of pressure displacement using a force platform (AccuswayPLUS, AMTI, USA) sampled at 50 Hz and low-pass filtered with a cutoff frequency at 5 Hz. The standard deviation was computed from the displacement of the center of pressure for the anterior-posterior axis, which provides a measure of the width of amplitude variability in this axis. The bodyweight of each participant was measured using the same platform.

3. Design and Procedure

Participants were instructed to stand upright on the force platform, with bare feet together, arms relaxed along the trunk and to look at the monitor to start the recording session. The recording session comprised the presentation of three gray screens with a central white fixation cross displayed for 48 s each; and the two sets of pictures (control and threat), also displayed for 48 s each, totalizing 240 s. Within each set, individual pictures were presented for three seconds with no interval between them. The control set preceded the threat set (Figure S1).

At the end of the experiment, participants filled-in the Trauma History Questionnaire (THQ), Posttraumatic Stress Disorder Checklist for DSM-IV (PCL-C for DSM-IV). Then, participants answered the following question “Do you play video games with violent contents (people using firearms)?”. The alternatives were “never”, “sometimes”, “often”, and “almost always”.

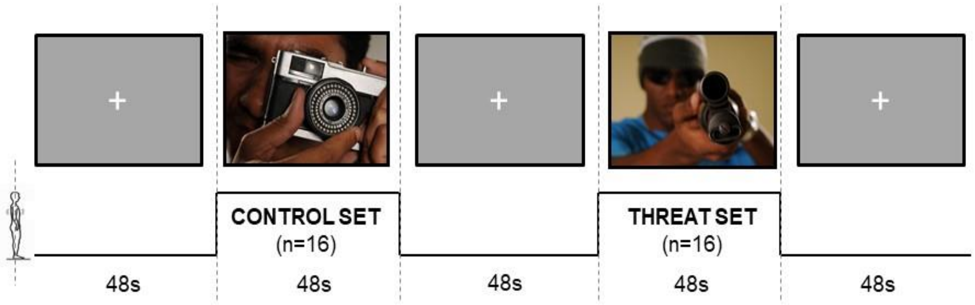

Supplementary Figure 1. Schematic representation of the experimental design. Participants stood upright on the force platform and were instructed to look at the monitor. The recording session started with a gray screen followed by the control set; a second gray screen was presented before the threat set; a third gray screen ended the session.
